# Effect of the MyDéfi Smartphone Application on Binge Drinking Among University Students: Protocol of a Double‐Blind Multicenter Prospective National Randomized Controlled Trial Using Phosphatidylethanol as a Biomarker—The SMARTBINGE Trial

**DOI:** 10.1002/mpr.70014

**Published:** 2025-04-01

**Authors:** Camille André, Pierre Sauton, Méléna Dreinaza, Momar Diouf, Sandra Bodeau, Margaret Martinetti, Raphaël Trouillet, Clara de Groote, Jean‐Louis Nandrino, Adèle Alexandre, Farid Benzerouk, Fabien Gierski, Pascal Perney, Laure Grellet, Judith André, Mickael Naassila

**Affiliations:** ^1^ Groupe de Recherche sur l’Alcool & les Pharmacodépendances INSERM UMR 1247 Université de Picardie Jules Verne Amiens France; ^2^ Department of Statistics Amiens Hospital University Amiens France; ^3^ Department of Clinical Pharmacology Amiens University Medical Center Amiens France; ^4^ University of Paul Valéry Montpellier 3 Montpellier France; ^5^ Laboratory SCALab UMR CNRS 9193 University of Lille Villeneuve d'Ascq France; ^6^ Cognition Health Society Laboratory (C2S ‐ EA 6291) University of Reims Champagne Ardenne Reims France; ^7^ Addictions Department CHU Caremeau University of Montpellier Montpellier France; ^8^ Sexology Office Montpellier France

**Keywords:** binge drinking, digital intervention, e‐health, mobile application, phosphatidylethanol

## Abstract

**Objective:**

The purpose of this paper is to describe a study protocol of a clinical trial exploring the effectiveness of the new mobile application MyDéfi proposing personalized feedback, on both alcohol consumption and quality of life, as well as the blood alcohol exposure biomarker phosphatidylethanol, in university students displaying binge drinking behavior.

**Methods:**

This prospective national multicentric randomized, two‐arm (1:1), double‐blind controlled trial will recruit 628 students (aged 18–25 years) with binge drinking behavior. Participants will be randomized in the “intervention” group (personalized feedback) or the “control” group (generic feedback) and will undergo four monthly visits. Monthly dried blood spot sample for measuring phosphatidylethanol concentration and online questionnaires will be collected. Our primary objective is to assess the reduction weekly standard drinks, through self‐report gathered via MyDéfi. Secondary objectives will evaluate the application's impact on the dosage of phosphatidylethanol blood concentration and on quality of life”.

**Results:**

Recruitment started in March 2024 and will end in March 2026.

**Conclusion:**

This study aims to determine the effectiveness of two versions of the same mobile application (generic vs. personalized feedback) on alcohol consumption in students displaying binge drinking behavior. The effectiveness of the application will be measured, with a secondary objective of quantifying phosphatidylethanol. Our study will open new perspectives on the use of digital interventions for students who do not actively seek treatment.

**Trial Registration:**

Trial registration number (NCT06084832), the date of registration (10th October 2023) and when this was done (16th October 2023). https://clinicaltrials.gov/study/NCT06084832

## 1 Introduction

Binge drinking (BD) produces several harmful consequences specifically among adolescents and young adults (Rolland et al. 2017; Streel et al. 2019) and is particularly entrenched in student culture (Kypri et al. 2005; Merrill and Carey 2016) as 33.9% of young European adults aged 20‐24 display BD behavior (World Health Organization (WHO), 2018). In France, few studies on BD are available on college students, with varying prevalence rates (from 38% to 77.4% in men and from 23% to 59.3% in women) (Lorente et al. 2003; Martha et al. 2009; Tavolacci et al. 2016).

Adolescents and young adults are particularly vulnerable to the neurotoxicity of alcohol. Numerous clinical and preclinical studies have demonstrated brain and cognitive impairments, as well as cellular mechanisms underlying learning and memory processes (Drissi et al. 2020; Vilpoux et al. 2022) (Gierski et al. 2020; Guerri and Pascual 2010; Townshend and Duka 2005). The consequences on cognition and quality of life are manyfold: sleep and memory problems, emotional difficulties, poorer school results, unsafe sexual relationships and violence. In addition, BD is also associated with an increased risk of developing alcohol use disorders (AUD) (Tavolacci et al. 2019).

As BD during adolescence is a risk factor for AUD, prevention is crucial, particularly through primary prevention interventions among adolescents. However, there is a clear need to extend prevention strategies to secondary prevention for young people with existing drinking problems (Murphy et al. 2001). This targeted secondary prevention can be achieved through Screening, Brief Intervention, and Referral to Treatment (SBIRT). SBIRT interventions (Babor and Higgins‐Biddle 2001), are psychosocial counseling sessions lasting between 5 and 30 min and including screening, feedback and motivational interviewing strategies. They provide early intervention for drinkers who exceed WHO guidelines for alcohol consumption, but are not alcohol‐dependent and do not seek to reduce this risky behavior (Nilsen 2010). This approach is based on the theory of planned behavior (TPB). This theory suggests that an individual's intentions are the main determinants of their behavior and aims to identify the individuals and environmental factors that influence these intentions (Ajzen 1991; Webb et al. 2010).

However, obstacles can hinder the SBIRT deployment in the treatment of AUD: time, access to healthcare professionals, stigmatization. These barriers tend to be reduced by brief intervention (BI) practiced through e‐intervention called brief e‐health interventions (e‐BI). E‐health refers to health services and information provided via the internet and related technologies (computer, mobile phone). It enables the implementation of an e‐intervention with a number of benefits: anonymity, ease of access, increased effectiveness of self‐management (Kapitány‐Fövény et al. 2022). Indeed, these characteristics correspond more closely to students' requirements (Buscemi et al. 2010) and can motivate students to seek help from the healthcare system when they need it.

Mobile applications are an avenue worth exploring as e‐BI for reducing alcohol consumption among students, with 93% of 18–24‐year‐olds in France owning a smartphone in 2023 (Center de Recherche pour l’Étude et l’Observation des Conditions de vie (CRÉDOC), 2024). Indeed, the use of smartphones enables identification of factors associated with BD in real time, in accordance with the TPB, using, for example, a daily consumption diary or personalized feedback based on the patient's habits. Some mobile applications, such as the MyDéfi application developed by *Extellient*, send personalized notifications to users based on their daily consumption, previously filled in the consumption diary. The MyDéfi application has been developed by specialists in addictions medicine and allows the users to track their consumption over a 12‐week program. The personalized messages, regarding alcohol consumption, drinking risk level and the evolution of alcohol consumption, have been created based on SBIRT and motivational intervention (Hien et al. 2022). Personalized feedback, adapted to the characteristics and behavior of the user, has already proved to be an effective e‐BI (Song et al. 2019; Tansil et al. 2016; Teeters et al. 2018). The importance of a relevant and appropriate “control" group in clinical trials has been emphasized in order to be able to conclude on the effectiveness of personalized returns on BD behavior but also to limit the digital placebo effect (Torous and Firth 2016a; Williamson et al. 2022). Indeed, to conclude on the effectiveness of personalized feedback, we chose to compare this mobile application (with personalized feedback) with a second version of the same application, with generic feedback. With these two arms of the clinical study differing from each other only in the presence or absence of personalized feedback, we can conclude that personalized feedback is effective. Thus, the digital placebo effect (defined as the therapeutic effect resulting from the simple use of the smartphone without any real effect of the mobile application) will be limited (Torous and Firth 2016b).

Moreover, to corroborate the effectiveness of drinking diaries, self‐questionnaires and personalized feedback on reducing alcohol consumption, it is worth combining them with the measurement of a reliable and objective biological marker of alcohol consumption (Fakhari and Waszkiewicz 2023; Grüner Nielsen et al. 2021; Tawiah et al. 2022). Phosphatidylethanol (PEth) is a highly sensitive and specific biomarker with a long half‐life (3–5 days) (Wurst et al. 2010). This abnormal phospholipid is formed by transphosphatidylation of precursor phospholipids by phospholipase D only in the presence of ethanol. It is not degraded by any enzymatic system, persisting for 3–4 weeks in the erythrocyte membrane (Alling et al. 1983; Varga and Alling 2002). Among these 48 homologs, the 16:0/18:1 and 16:0/18:2 forms are the most abundant (Helander and Zheng 2009). These high levels of specificity and sensitivity are used to divide drinkers into three groups, based on their alcohol consumption in the last month: a blood concentration of less than 20 ng/mL means abstinence or low consumption (less than 20 g of pure ethanol per day); a blood concentration of between 20 and 200 ng/mL means average consumption (between 20 and 40 g per day); a blood concentration of more than 200 ng/mL means excessive consumption (more than 40 g per day) (Simon 2018; Ulwelling and Smith 2018).

Finally, the necessity of developing new therapeutic strategies to manage a population of students who practice BD, presenting both a risk of developing cognitive complications and a lower quality of life, is crucial, especially as this population only seeks help from healthcare professionals at a late stage. An e‐BI approach using a mobile application with personalized feedback therefore seems appropriate for this population, which has difficulty accessing the medical profession. The self‐reported alcohol consumption and behaviors could be corroborated using PEth as a sensitive and specific biomarker of consumption. Our aim is to determine the effectiveness of personalized feedback via the MyDéfi mobile application in reducing alcohol consumption in a BD student population. The second objective of this study is to demonstrate in parallel the correlation between a reduction in alcohol consumption recorded via a consumption diary, associated with changes in blood PEth concentration and an improvement in quality of life.

## 2 Methods and Analysis

### 2.1 Study Design and Setting

The SMARTBINGE study is a prospective multicentric randomized (1:1), controlled, double‐blind trial with a 3 months follow‐up and two parallel arms compared to each other. The intervention group will have access to the MyDéfi mobile application with personalized feedback tailored from their diary daily alcohol consumption. The control group will have access to MyDéfi but, although with the same number of messages, with generic feedback without adaptation from their diary daily alcohol consumption (e.g. a “placebo” application). Since students are unable to distinguish whether the messages, they receive are personalized, our protocol meets the criteria for a double‐blind design. Six hundred and twenty‐eight university students will be recruited from four French universities: Amiens (University of Picardie Jules Verne), Reims (University of Reims Champagne Ardennes), Lille (University of Lille) and Montpellier (University Paul Valéry Montpelier 3) (https://clinicaltrials.gov/study/NCT06084832).

### 2.2 Eligibility Criteria

The inclusion criteria are as follow: the participants must be university students between 18 and 25 years old, have a score greater or equal to 24 to the BD score (J. M. Townshend 2002), a score greater or equal to 3 to the first 3 items of the Alcohol Use Disorders Identification Test (AUDIT) questionnaire, have a BD behavior (at least one occasion with 6 or more drinks (≥60 g pure ethanol) in a single occasion during the last 3 months), consent to be included in the study and be affiliated to social security (having an identified social security number). It is a requirement for a participant to be affiliated to the social security system, because in France, any biomedical research on a person who is not affiliated to that system or is a beneficiary of it is prohibited. Psychologists and neurosciences researchers, trained to good clinical practices, will ensure recruitment.

The exclusion criteria are as follow: not having a smartphone with an Apple or Android system, having previously used the MyDéfi mobile application, declaring a psychiatric or neurological condition (self‐reported data), being a pregnant, parturient or breastfeeding woman; or being subject guardianship, curatorship or restricted under public law. Being subject to guardianship, curatorship, or restrictions under public law refers to legal measures applied to individuals who are deemed unable to fully manage their personal, financial, or legal affairs due to a medical, psychological, or cognitive condition. Persons who are in one of these cases are unable to give consent for themselves and thus it is an exclusion criterion.

### 2.3 Recruitment

University students, from the four participating universities, will be proposed to join the SMARTBINGE study through leaflets displayed in university facilities or via a first email on their institutional email address. In this mail, a link to access the first questionnaires on online LimeSurvey platform will allows them to answer the AUQ (Alcohol Urge Questionnaire), AUDIT, DDQ (Daily Drinking Questionnaire), Drug Use Questionnaire, UPPS‐S (Urgency, Premeditation, Perseverance, and Sensation seeking sale) and TCI‐125 (Temperament and Character Inventory‐125) questionnaires. Based on their responses and if they meet the inclusion criteria, participants are selected and invited through email to the inclusion visit by the investigator of their university (using an email address specifically created for the study).

### 2.4 Randomization, Concealed Allocation and Blinding

During the inclusion visit, participants are randomized 1:1 into the control or intervention group using the Ennov Clinical software set up by the Amiens University Hospital data manager. Randomization will be done by minimization stratified. During the process, participants' inclusion and exclusion criteria are checked, their birth date and initials will be registered and then, patients will be randomized. For each inclusion, the Ennov Clinical software generates a five numbers inclusion code to ensure data tracking in an anonymous manner. For each participant, the result of the randomization and the inclusion code will be transferred to the MyDéfi developers by the Ennov Clinical software, to ensure double‐blind status. Neither the participants nor the investigators know the allocation of the groups.

### 2.5 Interventions

Intervention group will have the intervention application with personalized feedback tailored from their diary daily alcohol consumption, while the control group will have the placebo application with generic feedback. More details on these two versions can be found in the section “MyDéfi smartphone application: consumption diary”.

We will limit the number of missing data by sending reminders through both emails and smartphone app messages to prompt participants to complete questionnaires, consumption diaries, and sample collections on time. Participants who cannot be available on a follow‐up visit are provided during a prior visit with the necessary material to perform micro‐blood sampling at home (HemaXis DB 10 kit, label etc.), and are asked to bring the sample with them on the next visit. Participants are trained to correctly use the HemaXis DB 10 kit during the first visit.

As recommended by our clinical research and innovation services of the CHU Amiens‐Picardie (Leadpartner of the SMARTBINGE trial), all participants are compensated up to 100€, in the form of gift cards usable at various major retail chains (20€ at M0, 20€ at M1€ and 60€ at M3). Any participant may, at any time, withdraw prematurely from the study without prejudice to his/her subsequent treatment.

#### 2.5.1 Socio‐Demographic Data

During the initial approach, along with the online questionnaires on the online LimeSurvey platform, we will collect age, sex of birth, gender, weight and university course.

#### 2.5.2 Online Questionnaires

The parameters to be studied in each self‐questionnaire are presented in Table 1. Alcohol consumption will assess alcohol consumption using 4 validated questionnaires: 8‐items AUQ, AUDIT‐long, AUDIT‐3R, DDQ (Collins et al. 1985; Cortés‐Tomás et al. 2017; Mehrabian and Russell 1978; J. M. Townshend 2002). The use of other drugs will be evaluated by 3 validated questionnaires: Questionnaire on Drug Use, simplified Fagerström questionnaire and CAST (Cannabis Abuse Screening Test) (Heatherton et al. 1991; Legleye et al. 2007; Morgan and Grube 1991). Drinking motives will be assessed using the Drinking Motives Questionnaire Revised Short Form (DMQ‐R SF) (Kuntsche and Kuntsche 2009).

**TABLE 1:** Online auto‐questionnaires assessing alcohol and drugs consumption, personality and quality of life completed by study participants.

Questionnaires	Assessment	Number of items	References
Alcohol consumption			
AUQ	Frequency and quantity over the past 6 months of alcohol consumption	8	(Mehrabian and Russell 1978; J. M. Townshend 2002)
AUDIT‐long	Detection of AUD over the past year	10	(Babor and Higgins‐Biddle 2001)
AUDIT‐3R	Detection of AUD over the past year	3	(Cortés‐Tomás et al. 2017)
DDQ	Estimate a typical number of drinks consumed each day of the week over the previous 30 days	12	(Collins et al. 1985)
Use of other drugs			
Questionnaire on drug use	Use of tobacco and 12 illicit substances consumption	38	(Morgan and Grube 1991)
Simplified Fagerström questionnaire	Tobacco consumption	3	(Heatherton et al. 1991)
CAST	Cannabis consumption	6	(Legleye et al. 2007)
Personality			
DMQ‐R SF	Identification of motivations for consumption over the past 12 months according to 4 motivational components: Reinforcement, coping, social, and conformity	12	(Kuntsche and Kuntsche 2009)
RCQ	Motivation to change alcohol, cannabis, and other drug consumption habits	36	(Rollnick et al. 1992)
Rosenberg self‐esteem scale	Level of self‐esteem	10	(Avison and Rosenberg 1981)
TCI‐125	Novelty seeking and harm avoidance subscales	40	(Chakroun‐Vinciguerra et al. 2005)
UPPS Impulsive behavior scale	Level of impulsivity in four distinct facets: Urgency, premeditation, perseverance, and sensation seeking	20	(Billieux et al. 2012)
GAD‐7	Screening of anxiety disorder	7	(Spitzer et al. 2006)
PHQ‐9	Depression	9	(Spitzer 1999)
Quality of life			
AQoLS	Impact of alcohol on the patient's life over the past 4 weeks	34	(Luquiens et al. 2015)
BYAACQ	Alcohol‐related consequences and identify at‐risk students	24	(Kahler et al. 2005; Read et al. 2006)
APT	In a simulated market, purchase of alcohol at different prices	15	(Martinetti et al. 2019)
APT choice	In a simulated market, purchase of alcohol and alternative non‐alcoholic beverage at different prices	15	(Martinetti et al. 2019)

Abbreviations: APT, alcohol purchase task; APT Choice, alcohol purchase task with non‐alcoholic beverage choice; AQoLS, alcohol quality of life scale; AUQ, alcohol urge questionnaire; AUDIT, alcohol use disorders identification test; BYAACQ, brief young adult alcohol consequences questionnaire; CAST, cannabis abuse screening test; DDQ, daily drinking questionnaire; DMQ‐R SF, drinking motives questionnaire revised short form; GAD‐7, generalized anxiety disorder assessment; PHQ‐9, patient health questionnaire; RCQ, readiness to change questionnaire; TCI‐125, temperament and character inventory‐125; UPPS, urgency, premeditation, perseverance, and sensation seeking.

Drinkers psychological and behavioral characteristics of consumption will be evaluated with 6 questionnaires. Readiness to change will be assessed using the Readiness to Change Questionnaire (RCQ) (Rollnick et al. 1992), self‐esteem, using the Rosenberg self‐esteem scale (Avison and Rosenberg 1981), temperament and Character with the Temperament and Character Inventory (TCI) (Chakroun‐Vinciguerra et al. 2005). Impulsivity will be assessed by the UPPS‐S Impulsive Behavior Scale (Billieux et al. 2012), anxiety with the Generalized Anxiety Disorder Assessment (GAD‐7) (Spitzer et al. 2006) and depression, using the Patient Health Questionnaire (PHQ‐9) (Spitzer 1999).

Impact of alcohol on quality of life will be assessed using the Alcohol Quality of Life Scale (AQoLS) (Luquiens et al. 2015) while alcohol consequences in the young adult will be evaluated using the Brief Young Adult Alcohol Consequences Questionnaire (BYAACQ) (Kahler et al. 2005; Read et al. 2006). Finally, behavioral economics regarding alcohol will be assessed using the Alcohol Purchase Task (APT) and the Alcohol Purchase Task with Non‐alcoholic Beverage Choice (APT Choice) (Martinetti et al. 2019).

This study aims to demonstrate the efficacy of a digital intervention in reducing alcohol consumption and BD. Beyond this primary objective, it is worth exploring the intervention's effects on additional parameters that may be linked to changes in BD behavior and could help explain the app's effectiveness. To comprehensively assess these outcomes, a range of validated self‐report questionnaires have been selected. Alcohol consumption will be measured using four established tools (AUQ, AUDIT‐long, AUDIT‐3R, DDQ), while the use of other substances will be evaluated with three additional questionnaires (Drug Use Questionnaire, simplified Fagerström, CAST). Given the potential role of drinking motives in alcohol use, the DMQ‐R SF will be included. Psychological and behavioral factors that may influence drinking behavior, such as readiness to change, self‐esteem, temperament, impulsivity, anxiety, and depression, will be assessed using six validated scales (RCQ, Rosenberg Self‐Esteem Scale, TCI, UPPS‐S, GAD‐7, PHQ‐9). Additionally, the impact of alcohol on quality of life (AQoLS) and the consequences of drinking in young adults (BYAACQ) will be evaluated. Lastly, behavioral economic factors related to alcohol consumption will be explored using the APT and its variant incorporating non‐alcoholic beverage choices (APT Choice). By integrating these measures, we aim to not only assess the primary efficacy of the intervention but also gain insights into the mechanisms underlying its impact on drinking behavior.

#### 2.5.3 MyDéfi Smartphone Application: Consumption Diary

The free MyDéfi mobile application from the French Society of Alcohology (SFA), was developed as a SBRI (Screening and Brief Personalized Intervention) tool offering e‐BI using motivational techniques and cognitive‐behavioral therapies. With a 12‐week program, the application aims to assist excessive alcohol consumers in autonomously reducing their consumption through personalized coaching tailored to their daily progress. Two versions of MyDéfi were developed for this study: a version delivering personalized message tailored to participants alcohol daily consumption (“intervention” application designed for the “intervention” group) and a version delivering generic messages not tailored to participants alcohol daily consumption (“placebo” application designed for the “control” group).

**FIGURE 1:**
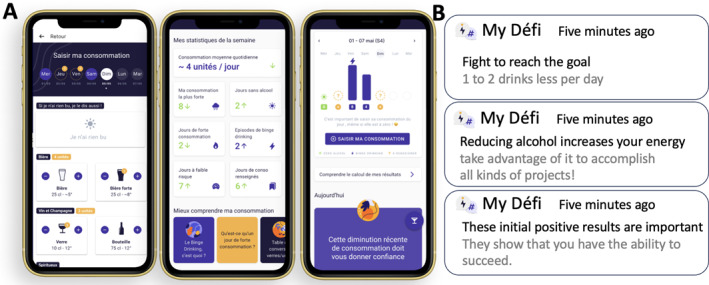
(A) Drinking diary on the MyDéfi application (in standard alcohol units of 10 g pure ethanol). (B) examples of notifications sent by MyDéfi.

For the “intervention” group, to provide tailored responses that mimic a face‐to‐face consultation, an algorithm‐based e‐BI takes into account: the evolution of consumption since the start of the program through the creation of a consumption agenda; the user's birth sex; and the program's timeline (beginning, middle, or end of the 12 weeks) (Figure 1). The personalized messages are not based on PEth data, as the measurements remain anonymous. The personalized messages are based on the consumption reported by users, episodes of BD, and the evolution of the consumption data recorded in the drinking diary. As a result, the messages vary and inform the user whether their consumption is risky, whether they have engaged in a BD episode, and how their consumption and BD behavior are evolving. The user always receives positive and encouraging messages, motivating them to change their behavior if it is risky, corresponds to BD, or based on their progress. For example, message at the start of the program after a reduction in consumption since the start of the protocol would be: “These first positive results are important, they show that you have the ability to succeed”. This message is highly personalized and cannot be offered at all times or to all users. For the “control” group, generic messages do not take any parameters into account. These messages are always valid, whatever the time of the program and therefore whatever the consumption trend. For example, “Don't drink one glass of alcohol after another as if it were water … it'll prevent a bad tomorrow and a black hole”. Because students cannot identify whether they are receiving personalized messages or not, the protocol is indeed double‐blind. For participants of both groups, alcohol consumption can be filled during the day or retrospectively the previous week on Sunday. Each message is sent via notifications, for that reason during the first visit (M0) the participants are asked to not disable notifications. The importance of keeping notifications enabled is reiterated at every monthly visits.

All data is anonymous, not collected for commercial or statistical purposes, and only accessible by the user. All data is encrypted and securely stored using shielding.

Study participants can enter the program with their five numbers inclusion code enabling them to access the version of MyDéfi assigned to them by randomization Ennov Clinical software (“control” or “intervention” group) to start the follow‐up. The email address and password requested are encrypted, inaccessible to investigators, and cannot be used to trace back to an ID. Those data simply allow the user to continue the program if they change phones.

The different versions of the application and the data extracted through MyDéfi are described in Supporting Inforamtion S1 (see “Description of the two versions of the MyDéfi smartphone application”). Examples of the drinking diary and notifications can be found on Figure 1.

#### 2.5.4 Micro Blood Sampling: Phosphatidylethanol Measure

**FIGURE 2:**
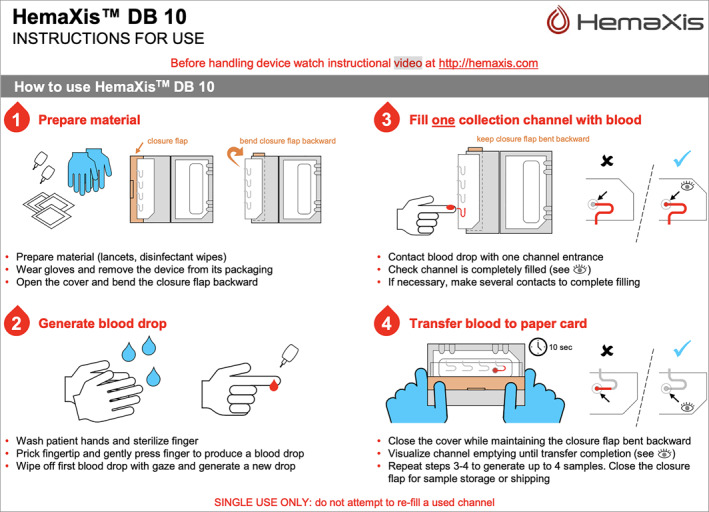
HemaXis DB 10 kits instruction for use.

The PEth exposure marker will be measured by dried blood spot (DBS) with HemaXis DB 10 kits with 4 self‐samples throughout the study: at the M0, M1, M2 and M3 inclusion visit. After washing hands and cleaning the sampling area on the finger with an alcohol‐free disinfectant wipe, 10 µL of blood is drawn from a channel and then deposited on blotting paper, forming a calibrated blood spot (Figure 2) (Hemaxis 2019). During M0, the investigator provided training in self‐sampling, with a video demonstration. All samplings are labeled with the participant's five number inclusion code and the sample date. Participants are informed during the inclusion visit that only the PEth exposure marker will be quantified in the blood, and that the data will be collected in accordance with French law n.78‐17 of January 6, 1978, and the “European Union Data Protection Directive” (95/46/EC of October 24, 1995).

All samples will be analyzed in the Pharmacology and Toxicology laboratory of CHU Amiens‐Picardie according to a validated analytical method (NF EN ISO15189 standard). Dried whole spot of PEth collected on HemaXis DB 10 kits (Ganz, Switzerland) will be determined by a HPLC‐MS/MS (high performance liquid chromatography tandem mass spectrometry) method (8060 LC‐MS/MS, Shimadzu, Marne‐la‐Vallée, France) (Spicher 2022). The analysis method can be found in Supporting Inforamtion S1 (see “Phosphatidylethanol sample analysis method”).

### 2.6 Outcomes

Our primary outcome is to demonstrate the effectiveness of a follow‐up with personalized feedback delivered via a mobile application in the intervention group in reducing the number of standard drinks per week compared to the control group by comparing the baseline M0 to the end of follow‐up M3.

Our secondary objectives are to compare between “intervention” group and “control” group a reduction in blood PEth concentration as well as a reduction of alcohol‐related impairments in several psychological dimensions (scores on personality DMQ‐R SF, RCQ, Rosenberg self‐esteem scale, GAD‐7, PHQ‐9) and quality of life questionnaires (AQoLS, BYAACQ, APT Choice) at the baseline M0 and at M3.

### 2.7 Participant Timeline

**FIGURE 3:**
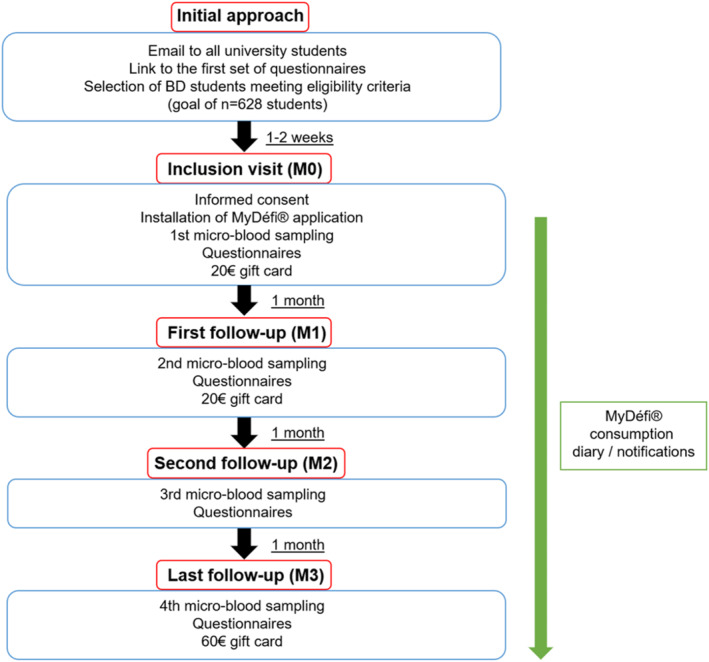
Study schedule of enrollment and assessments by time points. M0 = Month 0; M1 = Month 1; M2 = Month 2; M3 = Month 3.

The study schedule can be found on Figure 3 and the entire timeline can be found on Table 2. University students, from the four participating universities, will be proposed to join the SMARTBINGE study via a first email on their institutional email address. In this mail, a link to access the first questionnaires on online LimeSurvey platform will allows them to answer the AUDIT, AUQ, DDQ, Drug Use Questionnaire, UPPS‐S and TCI questionnaires in order to check whether students met the inclusion criteria (presence of BD behavior).

**TABLE 2:** Study schedule of enrollment, assessments, interventions and compensation by time points.

	Initial approach	Inclusion visit (M0)	First follow‐up (M1)	Second follow‐up (M2)	Last follow‐up (M3)
Eligibility screening	X				
Informed consent		X			
Randomization		X			
MyDéfi installation		X			
Self‐questionnaires					
Alcohol consumption					
AUQ	X	X	X	X	X
AUDIT (full scale)	X				
AUDIT‐3R		X	X	X	X
DDQ	X	X			X
Use of other drugs					
Drug use	X	X			X
Fagerström		X			X
CAST		X			X
Personality					
DMQR‐SF		X			X
RCQ		X			X
Rosenberg scale		X			X
TCI‐125	X				X
UPPS‐S	X	X			X
GAD‐7		X			X
PHQ‐9		X			X
Quality of life					
AQoLS		X			X
BYAACQ		X			X
APT/APT choice		X			X
Interventions					
PEth micro‐blood sampling		X	X	X	X
MyDéfi consumption daily diary		X	X	X	X
Compensation					
Gift card		X (20€)	X (20€)		X (60€)

Abbreviations: APT, alcohol purchase task; APT Choice, alcohol purchase task with non‐alcoholic beverage choice; AqoLS, alcohol quality of life scale; AUQ, alcohol urge questionnaire; AUDIT, alcohol use disorders identification test; BYAACQ, brief young adult alcohol consequences questionnaire; CAST, cannabis abuse screening test; DDQ, daily drinking questionnaire; DMQ‐R SF, drinking motives questionnaire revised short form; GAD‐7, generalized anxiety disorder assessment; PHQ‐9, patient health questionnaire; RCQ, readiness to change questionnaire; TCI‐125, temperament and character inventory‐125; UPPS, urgency, premeditation, perseverance, and sensation seeking.

Students meeting the inclusion criteria are contacted again by mail and are invited to the inclusion visit (M0). During the M0 visit, they will sign a consent form and will be randomized into one of the two groups after receiving an anonymous five‐number inclusion code. After downloading MyDéfi application, participants enter their anonymous five‐number inclusion code. This unlocks the version of the application assigned to them during randomization: intervention or placebo application version. Then, the participants will be trained to perform DBS sampling using an HemaXis DB 10 kit with a demonstration video and the guidance of an investigator. After sampling, students answer the M0 online questionnaires on LimeSurvey (see Table 2) at home just after blood sampling, to ensure consistency between the answers to the questionnaires and the blood sample.

During the M1 and the M2 visit, the participants will perform a new micro‐blood sampling using an HemaXis DB 10 kit and answer the AUDIT‐3R and AUQ questionnaires. Finally, for M3 visit, after micro‐blood sampling, participants will answer to the last set of questionnaires online (see Table 2).

### 2.8 Sample Size

Based on literature (Gierski et al. 2017), the sample size calculation assumed a standard deviation of 8 weekly number of drinks following Gierski's results on students with BD behavior. The expected outcome in each group drew from two studies: Bertholet's study on students with unhealthy alcohol use and Kaner's meta‐analysis. Bertholet's team anticipated reductions of 2 drinks per week in the intervention group (app users) and 0.39 drinks in the control group (Bertholet et al. 2023). Kaner's meta‐analysis of the comparison between digital versus no or minimal intervention showed a difference of 22.84 g of alcohol per week, thus the goal at M3 is to observe a reduction difference of 2 drinks (Cohen's effect size *d* = 0.25) from the mean number of drinks per week between the two groups (Kaner et al. 2015). Assuming a two‐sided alpha risk of 5% and 80% power, 253 participants per group will be needed. Considering an estimated drop‐out rate of about 20%, a total of 628 participants are necessary for the initial recruitment.

### 2.9 Data Management and Statistical Analysis

Participant data will be entered by the investigators into a pseudonymised computer database secured by passwords and centralized at the Amiens University Hospital, to which only the investigators will have access. The database will then be frozen at the end of the study.

For the primary outcome, the reduction in weekly number of drinks will be compared between the two arms using a mixed‐effects analysis of covariance (ANCOVA) model with adjustment for baseline weekly number of drinks and randomization stratification criteria (sex and center). The center will be treated as a random effect.

For the secondary outcomes, the evolution of quality of life, which is collected via questionnaires, will be compared between the two groups using a mixed‐effects ANCOVA model with adjustment for baseline quality of life and randomization stratification criteria (sex and center). The center will be treated as a random effect. Blood PEth concentration will be compared between the two groups using a mixed‐effects ANCOVA model adjusting for initial concentration and stratification factors. Various models will be used to assess the intervention effect throughout the follow‐up period and to check for potential differences in intervention effects across different follow‐up times.

The level of statistical significance chosen for all measures is *p* < 0.05. The software used for data processing will be SPSS. Using a multiple imputation technique, all analyses will be conducted on an intention‐to‐treat basis, including all randomized participants (Full Analysis Set). Intermediate missing data will be handled using maximum likelihood estimation. An analysis based on the Last Observation Carried Forward (LOCF) method will be performed as a sensitivity analysis. We expect a low rate of missing data (< 2%), these methods should be sufficient. In practical term to limit attrition and missing data, we will: compensate participation with 100€ over 3 months (the duration of the study); send reminders by e‐mail to encourage participants to complete questionnaires, consumption diaries and samples on time. Previous studies showing high completion rates (> 90%, e.g. in Bertholet's team work (Bertholet et al. 2023)).

## 3 Results

Since this is a study protocol, we are still in the recruitment phase, which began in March 2024 and will end in March 2026. To date, 116 participants have already been recruited, 39 of whom have completed their follow‐up. These first participants have allowed us to refine our forecast for the attrition rate. Current data show a drop‐out rate of around 10%, that can be attributed to the on‐site visits. This feature increases the power of the study to 85%. The results of the study will be published at a later date.

## 4 Discussion

Binge drinking is prevalent among university students. In general, student populations do not seek medical help. New technological tools to facilitate dematerialized and anonymous access are a necessity in terms of public health to reach this vulnerable population. The main purpose of the SMARTBINGE study, a prospective, randomized, double‐blind, multi‐center clinical trial is to demonstrate the efficacy of personalized feedback delivered via the MyDéfi mobile application in reducing the number of standard drinks per week in the “intervention” group of young adult students presenting a BD behavior.

To limit BD dangerous practice, a number of studies have tested the effect of mobile e‐health applications via smartphone use, which is widespread among this vulnerable population. Nevertheless, a number of biases have been identified in previous clinical studies studying mobile applications. Williamson's team's systematic review of the literature raised the issue of the lack of evidence supporting the hypothesis of the effectiveness of personalized notifications on drinking behavior, pointing to a number of pitfalls (Williamson et al. 2022). Yet this type of BI is preferred by users (Perski et al. 2018) and therefore potentially more effective. The authors highlight the fact that the randomized double‐blind studies, under‐represented in the literature, are interested in the impact of an app in general, and not the additional impact of personalized notifications included in an app, advising that each e‐BI should be isolated from a digital placebo effect (Torous and Firth 2016a). Indeed, in most studies on the effect of an application, the “control” group uses either the usual treatment (Gustafson et al. 2014), or a web page containing normative information (Bertholet et al. 2023; Gonzalez and Dulin 2015; Oldham et al. 2024), or nothing at all (Bertholet et al. 2015). The importance of a good “control” group is crucial, and while the examples cited can be used to determine the effect of an application, they cannot be used to determine the additional effects discussed above. Our study rectified this shortcoming by comparing two versions of the same mobile application in two separate randomized double‐blind arms: the effect of a “treatment" (intervention) diary mobile application with personalized feedback versus a “placebo" (control) diary mobile application with generic feedback.

Another limitation of studies using a consumption diary pointed out by Williamson's team (Williamson et al. 2022) lies in the subject's self‐reported consumption, overcome by measuring Peth in this study. Our secondary objectives are to seek a reduction of PEth whole blood concentration. As far as we know, this protocol is the first to correlate application mobile use with an objective measurement of last month's alcohol consumption, although blood PEth concentrations have already been correlated with BD practices, self‐reported (Chen et al. 2023; McLaughlin et al. 2022) or measured via the AUDIT score (Piano et al. 2015). The concentration of PEth in whole blood will be determined using a sensitive (LOQ = 9 ng/mL) and reproducible HPLC‐MS/MS method, with a simple preparation technique, equivalent to recently published HPLC‐MS/MS techniques (Kechagias et al. 2015; Nalesso et al. 2011; Zheng et al. 2011), fully validated in accordance with international guidelines (European Medicines Agency (EMA), 2015; “Scientific Working Group for Forensic Toxicology (SWGTOX) Standard Practices for Method Validation in Forensic Toxicology,” 2013). Furthermore, the in vitro instability of this molecule is resolved by using a DBS sampling device on blotting paper, allowing non‐invasive self‐sampling, and therefore not requiring medical staff, facilitating transport and extending the storage time of samples (Faller et al. 2013; Kummer et al. 2016).

Finally, these longitudinal studies are subject to the phenomenon of attrition, which we are trying to limit by proposing compensation for participants from M0 to M3. The 3‐month duration is typical for this type of study (ranging from 2 weeks to 1 year) (Williamson et al. 2022). However, it will be necessary to study the effect of MyDéfi over the long term (beyond 3 months).

Taking into account the recommendations and limitations of previous clinical studies, the design of our study sought to limit the numerous biases usually found in this type of study, by a single mobile application with two versions with a real placebo application (a “placebo” version with a diary with generic feedback vs. a “intervention" version with a diary with personalized feedback) while correlating the declarative consumption results with an objective biomarker and a measure of the many potential beneficial effects associated with a reduction in BD. If our study confirms the hypothesis that a e‐BI with personalized feedback reduces alcohol consumption, the MyDéfi mobile application specializing in BD could be recommended to provide ongoing, tailor‐made interventions combined with the advantages of e‐health, close to students' requirements (Buscemi et al. 2010; Kapitány‐Fövény et al. 2022), reaching this vulnerable population.

### 4.1 Ethics and Dissemination Policy

This study received approval from the ethics committee of Nantes Ouest IV (reference number 2023‐A01032‐43) on September 28th 2023, covered by civil liability insurance, in accordance with the provisions of article L1121‐10 of the Public Health Code. The data recorded during this research will be processed by the *Direction de la Recherche Clinique et de l’Innovation* department (DRCI, CHU Sud Amiens‐Picardie, Amiens, France) in accordance with the French Data Protection Act no. 78‐17 of 6 January 1978, as amended by Act 2004‐801 of 6 August 2004, and the General Data Protection Regulation (RGPD). This search has been registered on http://clinicaltrials.gov/. The study promoter ensured that each person participating in the research will give written informed consent. Results will be disseminated through open‐access, peer‐reviewed journal articles, conferences presentations and clinically focused workshops. A summary of the trial results will be made available to the participants on demand.

## Author Contributions

**Camille André:** data curation, investigation, visualization, writing – original draft, writing – review and editing. **Pierre Sauton:** data curation, investigation, project administration, visualization, writing – original draft, writing – review and editing. **Méléna Dreinaza:** investigation, data curation, visualization, writing – original draft, writing – review and editing. **Momar Diouf:** formal analysis, writing – review and editing, conceptualization, methodology. **Sandra Bodeau:** methodology, resources, project administration, supervision, validation, writing – review and editing. **Margaret Martinetti:** conceptualization, methodology, supervision, validation, writing – review and editing. **Raphaël Trouillet:** conceptualization, project administration, resources, supervision, validation, writing – review and editing. **Clara de Groote:** investigation, writing – review and editing. **Jean‐Louis Nandrino:** conceptualization, project administration, resources, supervision, validation, writing – review and editing. **Adèle Alexandre:** writing – review and editing. **Farid Benzerouk:** supervision, validation, writing – review and editing. **Fabien Gierski:** conceptualization, methodology, project administration, resources, supervision, validation, writing – review and editing. **Pascal Perney:** conceptualization, methodology, resources, software, supervision, validation, writing – review and editing. **Laure Grellet:** software, writing – review and editing. **Judith André:** conceptualization, data curation, supervision, validation, writing – review and editing, visualization. **Mickael Naassila:** conceptualization, funding acquisition, methodology, project administration, resources, supervision, validation, writing – review and editing.

## Conflicts of Interest

Laure Grellet and Pascal Perney are the co‐founders of the MyDéfi application which is used for the research project. They are not involved in student inclusion neither in results analysis. There are no other conflicts of interest to declare.

## Supporting information

Supporting Information S1

## Data Availability

Data sharing is not applicable to this article as no new data were created or analyzed in this study.
