# Supplementary material for: Effect of the MyDéfi Smartphone Application on Binge Drinking Among University Students: Protocol of a Double‐Blind Multicenter Prospective National Randomized Controlled Trial Using Phosphatidylethanol as a Biomarker—The SMARTBINGE Trial
Source: Int J Methods Psychiatr Res. 2025 Apr 1;34(2):e70014. doi: 10.1002/mpr.70014 (PMC11959157; doi:10.1002/mpr.70014)
Supplement: Supplementary file 1 — Supporting Information S1 [file MPR-34-e70014-s001.docx]

**Supplemental data**

**Description of the two versions of the MyDéfi® smartphone application**

The 3,000 messages and notifications delivered by the e-BI were specifically adapted in both content and style to young consumers (aged ≤ 25 years). Each day, the user receives specific messages that depend on the different data previously mentioned allowing a very personalized coaching:

(1) Advice on ways to reduce consumption. This reduction should be gradual to make it easy for the user to implement and to avoid withdrawal symptoms. Based on the reported consumption patterns, the proposed program can be automatically adjusted, without user intervention; switching from the "reducing binge drinking" program to the "reducing consumption" program (or vice versa). This program switch allows for ongoing assistance to students, even if they change their consumption pattern.

(2) To empower users, bibliographic information on alcohol toxicity, particularly BD: somatic, psychological, social, professional, family, and emotional complications are discussed, along with information on the expected benefits of reducing consumption.

(3) Motivational messages to reinforce the user's intrinsic motivation for change and strengthen their sense of self-efficacy. All these messages depend on consumption evolution but also on the program's stage (beginning, middle, or end of the 12 weeks).

(4) Messages aimed at encouraging adherence, i.e., regular use of the application and retention in the cohort.

Furthermore, these various messages and the completion of the consumption agenda are reinforced by notifications and various emoji visuals. Many visuals and explanations about its consumption are offered to users and the delivered messages vary throughout the program. Finally, to maintain a sense of novelty and personalized responses throughout the 12-week program, the user never receives the same message twice.

For the “control” group, the coaching is not personalized but proposes nevertheless (1) Advice on ways to reduce consumption; (2) Bibliographic information; (3) Motivational messages but not depending on participant alcohol consumption; (4) Messages aimed at encouraging adherence.

The following data are extracted through MyDéfi®: number of BD days per week, number of heavy drinking days per week (characterized by consuming more than six standard drinks for men / more than four standard drinks for women), total consumption per week, number of days with zero consumption, number of low-risk drinking days and number of standard alcohol units reduced per week / cumulative number of standard alcohol units reduced since the start of the program. All data is anonymous, not collected for commercial or statistical purposes, and only accessible by the user. All data is encrypted and securely stored using shielding.

**Phosphatidylethanol sample analysis method**

Briefly, the whole blood spot on blotting paper is cut in a circular pattern using a hammer and mandrel (diameter 7 mm). The PEth is extracted for 1 hour with 250 µL of methanol containing the deuterated internal standard (PEth-d_5_) (Sigma Aldrich, Saint-Quentin-Fallavier, France) at a concentration of 50 ng/mL. Then 150 µL of the extraction solution was placed in a vial and 5 µL was injected into the chromatographic system. Chromatographic separation was performed at 60°C on an Acquity UPLC BEH C18 1.7 µM (2.1 x 50mm, Waters™, Saint-Quentin-en-Yvelines, France). The column was eluted with a gradient of mobile phase A (A: ultrapure water/acetonitrile [30/70, v/v]) and mobile phase B (B: acetonitrile / isopropanol [10/90, v/v]) at a flow rate of 0.4 mL/min during a 10 min run. Data were acquired in multiple reaction monitoring (MRM) mode after ionization in negative electrospray ionization mode. MRM transitions used for 16:0/18:1 PEth and 16:0/18:2 PEth quantification were respectively: 701.4 -> 281.25 and 699.5-> 279.25. The LOQ is 9 ng/mL. Linearity of the method covers all consumer profiles from 0 to 1000 ng/mL. This sensitive and reproductible method was fully validated according to the guidelines issued by the European Medicine Agency (EMA) (‘Bioanalytical Method Validation - Scientific Guideline | European Medicines Agency (EMA)’, 2012) and the Scientific Working Group for Forensic Toxicology (SWGTOX) (Scientific Working Group for Forensic Toxicology, 2013).

**REFERENCES**

Bioanalytical method validation—Scientific guideline | European Medicines Agency (EMA). (2012, February 1). Retrieved 6 November 2024, from https://www.ema.europa.eu/en/bioanalytical-method-validation-scientific-guideline

Scientific Working Group for Forensic Toxicology. (2013). Scientific Working Group for Forensic Toxicology (SWGTOX) standard practices for method validation in forensic toxicology. *Journal of Analytical Toxicology*, *37*(7), 452–474. doi: 10.1093/jat/bkt054
